# Early discharge hospital at home as alternative to routine hospital care for older people: a systematic review and meta-analysis

**DOI:** 10.1186/s12916-024-03463-3

**Published:** 2024-06-18

**Authors:** Lulu Lin, Mengyuan Cheng, Yawei Guo, Xiaowen Cao, Weiming Tang, Xin Xu, Weibin Cheng, Zhongzhi Xu

**Affiliations:** 1 School of Public Health, Sun Yat-Sen University, Guangzhou, China; 2 Institute for Healthcare Artificial Intelligence Application, Guangdong Second Provincial General Hospital, Guangzhou, China; 3 Bouvé College of Health Sciences, Northeastern University, Boston, MA USA; 4 Institute of Global Health and Infectious Diseases, University of North Carolina at ChapelHill, Chapel Hill, NC USA; 5 School of Data Science, City University of Hong Kong, Hong Kong SAR, China; 6 Faculty of Health Sciences, City University of Macau, Macao SAR, China

**Keywords:** Early discharge hospital at home, Home care services, Older people, Meta-analysis

## Abstract

**Background:**

The global population of adults aged 60 and above surpassed 1 billion in 2020, constituting 13.5% of the global populace. Projections indicate a rise to 2.1 billion by 2050. While Hospital-at-Home (HaH) programs have emerged as a promising alternative to traditional routine hospital care, showing initial benefits in metrics such as lower mortality rates, reduced readmission rates, shorter treatment durations, and improved mental and functional status among older individuals, the robustness and magnitude of these effects relative to conventional hospital settings call for further validation through a comprehensive meta-analysis.

**Methods:**

A comprehensive literature search was executed during April–June 2023, across PubMed, MEDLINE, Embase, Web of Science, and Cumulative Index of Nursing and Allied Health Literature (CINAHL) to include both RCT and non-RCT HaH studies. Statistical analyses were conducted using Review Manager (version 5.4), with Forest plots and *I*^2^ statistics employed to detect inter-study heterogeneity. For *I*^2^ > 50%, indicative of substantial heterogeneity among the included studies, we employed the random-effects model to account for the variability. For *I*^2^ ≤ 50%, we used the fixed effects model. Subgroup analyses were conducted in patients with different health conditions, including cancer, acute medical conditions, chronic medical conditions, orthopedic issues, and medically complex conditions.

**Results:**

Fifteen trials were included in this systematic review, including 7 RCTs and 8 non-RCTs. Outcome measures include mortality, readmission rates, treatment duration, functional status (measured by the Barthel index), and mental status (measured by MMSE). Results suggest that early discharge HaH is linked to decreased mortality, albeit supported by low-certainty evidence across 13 studies. It also shortens the length of treatment, corroborated by seven trials. However, its impact on readmission rates and mental status remains inconclusive, supported by nine and two trials respectively. Functional status, gauged by the Barthel index, indicated potential decline with early discharge HaH, according to four trials. Subgroup analyses reveal similar trends.

**Conclusions:**

While early discharge HaH shows promise in specific metrics like mortality and treatment duration, its utility is ambiguous in the contexts of readmission, mental status, and functional status, necessitating cautious interpretation of findings.

**Supplementary Information:**

The online version contains supplementary material available at 10.1186/s12916-024-03463-3.

## Background

Hospital-at-Home (HaH) emerges as an innovative healthcare model, redefining the boundaries of hospital care by extending clinical management to the patient’s residence. Leveraging a meticulous blend of early discharge and admission avoidance strategies, HaH is driven by specialized teams that conduct comprehensive health and rehabilitative evaluations, either remotely or through home visits, ensuring the delivery of nuanced and patient-centric care [1].

The inception of HaH is particularly poignant against the backdrop of a burgeoning aging population, heralding a paradigm that fosters the efficient allocation of healthcare resources while accentuating the centrality of patient welfare [2]. It has garnered notable attention and application, particularly in the care of older adult patients grappling with a spectrum of conditions such as orthopedic anomalies [3] and chronic obstructive pulmonary disease (COPD) [4].

A synthesis of empirical explorations into HaH underscores its potential to recalibrate the cost-effectiveness landscape of healthcare delivery. The model, through its emphasis on early hospital discharge, appears to nurture an ecosystem that not only preserves but potentially enhances the quality of clinical outcomes [3, 5]. For example, a randomized controlled trial demonstrated that a short hospital stay followed by a well-managed home care program is as effective as a traditional 10-day hospitalization course. This approach not only reduces hospitalization costs but also fosters closer relationships between patients and their relatives [6].

However, the trajectory of HaH is not without its intersections of uncertainty and contention. Notable voices in the academic discourse have raised concerns regarding the sufficiency and robustness of evidence elucidating the comparative impacts of care environments on the rehabilitation outcomes of older individuals [7]. A nuanced examination of existing literature reveals a confluence of findings, where HaH programs, despite their transformative potential, echo with resonances of variability and ambiguity, particularly concerning readmission timelines [8].

In navigating these complexities, our study embarks on a systematic review and meta-analysis aimed at unraveling the comparative efficacies of early discharge HaH programs. Anchoring our inquiry are important metrics such as mortality rates, readmission frequencies, durations of treatment, and mental and functional statuses. Our exploration is channeled towards discerning the impact of these programs on older adults, aged 60 and above, within the architectural frameworks of traditional inpatient and HaH care paradigms. This endeavor is inspired by a commitment to enriching the empirical foundations that guide the optimization of HaH strategies in alignment with the evolving contours of patient needs and healthcare excellence.

## Methods

This systematic review and meta-analysis adhered to the Preferred Reporting of Items for Systematic Reviews and Meta-Analyses (PRISMA) guidelines, as detailed in Additional file 1: Table S1. The methodology employed in this review was guided by the Cochrane Handbook for Systematic Reviews of Interventions. This study was registered with PROSPERO (registration number: 321343). The protocol can be accessed at https://www.crd.york.ac.uk/PROSPERO/display_record.php?RecordID=321343.

### Search strategy and eligibility criteria

From April to June 2023, we conducted a rigorous search across multiple electronic databases to locate studies published subsequent to Gonçalves-Bradley DC’s 2017 review [9]. This encompassed peer-reviewed articles from databases such as PubMed, MEDLINE, Embase, Web of Science, and CINAHL. Additionally, our search was comprehensive, including grey literature such as abstracts and conference proceedings, and a manual review of references from relevant studies and key trial registries like ClinicalTrials.gov.

We used a combination of Medical Subject Headings (MeSH) and keywords relevant to hospital-sponsored healthcare services in the home setting. All studies considered were published in English. The search strategy, detailed in Additional file 1: Text S1, resulted in 5796 entries, shaping the pool from which we selected suitable literature for our review.

Upon the elimination of duplicate entries from the initial database search, two authors independently reviewed titles and abstracts for eligibility. Any discrepancies in the selection process were first resolved through discussion; if a consensus cannot be reached, a third author would arbitrate.

#### Eligibility criteria

Our review targeted studies that evaluated the efficacy or effectiveness of hospital-sponsored health care services provided in patients’ homes compared to usual in-hospital care. Eligible studies reported or permitted the extraction of raw data for one or more of our primary outcomes. We focused on studies involving older adults (aged ≥ 60 years) or those with a subgroup of individuals aged ≥ 60 for whom results were separately reported. We imposed no disease-specific restrictions to assess the home hospital programs’ efficacy across diverse health care needs. To ensure a thorough analysis, both randomized controlled trials (RCTs) and non-randomized controlled trials (non-RCTs) were included.

#### Inclusion criteria

Population: Individuals aged 60 years or older receiving health care services at home, who would otherwise require hospital care. Intervention: Health care services provided by physicians or nurses during acute or non-acute phases of illness at the patient’s home. Comparator: Standard inpatient hospital care.

#### Exclusion criteria

Excluded were studies focusing on outpatient care, residential care settings, or primarily involving patient self-care at home. Programs offering end-of-life care, social services (e.g., assistance with daily living), or transitional “hospital to home” care were also omitted. Review articles, commentaries, and study protocols were excluded due to the absence of outcome data.

#### Outcome measures

Primary outcomes included mortality rates during the study period or at specific time points, return hospital rates (admissions post-HaH or readmissions after inpatient care), functional ability measured by the Barthel Index, and quality of life assessed via standardized questionnaires like SF-36 or EQ-5D. Secondary outcomes encompassed cognitive function and depression levels.

#### Study selection

**Fig. 1:**
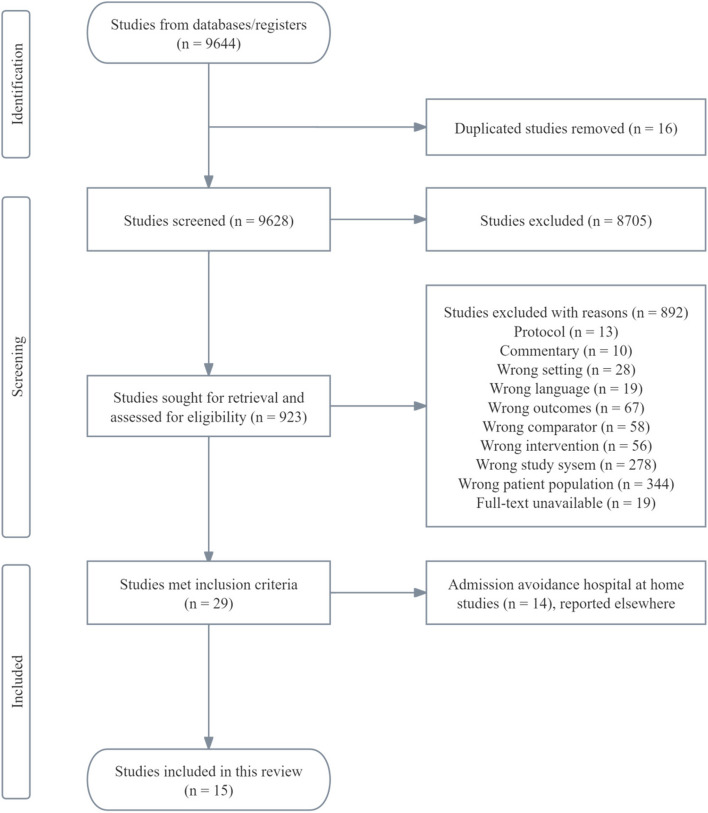
Flow diagram of study selection process

The selection process, documented via a PRISMA flow diagram (Fig. 1), began with removing duplicates from database searches. Two authors independently screened titles and abstracts for eligibility, with full-text articles reviewed for those preliminarily selected. Disagreements were resolved through consensus or consultation with a third author if necessary.

### Data extraction

Two independent reviewers are responsible for extracting relevant data from studies that meet the inclusion criteria. Information such as first author, publication year, geographical setting, study design, sample size, duration of follow-up, classification of caregivers, types of health services provided, utilization of telemedicine technologies, control groups, and outcomes measured were systematically documented using an electronic data collection form, accessible in the Additional file 1: Table. S2. In cases of missing or ambiguous data, inquiries were directed to the corresponding authors for clarification.

### Quality assessment

In assessing the quality of the included studies, we employed the Version 2 of the Cochrane risk-of-bias tool (RoB2 tool) [10] for randomized trials and the Risk Of Bias In Non-randomised Studies—of Interventions (ROBINS-I tool) [11] for non-randomized studies to evaluate potential biases systematically. Our assessment covered biases from the randomization process, deviations from intended interventions, missing outcome data, measurement of outcomes, and the reporting of results. Each domain of bias was judged according to the risk level: low, some concerns, or high. The overall quality of evidence for the outcomes reported in the studies was appraised using the RoB2 for RCTs and the ROBINS-I for non-RCTs and classified into three levels—high, some concerns, and low. Discrepancies in the quality assessment were resolved through discussion or by involving a third author for consensus. We visualized these assessments using Risk of Bias VISualization (robvis) tools [12] to aid in the clear presentation of our findings. Although certain domains within some studies raised “some concerns,” the collective evaluation of these studies generally indicates a low risk of bias.

### Statistical analyses

In conducting a meta-analysis, the conventional practice typically involves segregating RCTs from non-RCTs due to the inherent differences in study design and potential for bias. This separation is rooted in the aim to ensure the integrity and reliability of the analysis by comparing like with like. However, when such a distinction results in an insufficient number of studies within each category, the meta-analysis may face challenges related to statistical power. There is a trade-off between methodological purity and the practical necessity of accruing sufficient data to enable a meaningful analysis. To this end, main analyses were conducted by pooling RCT data and non-RCT data separately. For secondary analyses targeting specific diseases in this study, we have not conducted separate analyses due to an insufficient number of studies within each category, which could compromise the statistical power of the meta-analysis.

For datasets exhibiting homogeneity, a pooled meta-analysis was undertaken. This homogeneity was assessed according to the types of outcomes and their respective measurement time points across studies. Categorical outcomes such as mortality were presented as risk ratios with 95% confidence intervals (CIs), while continuous outcomes were expressed as mean differences (MDs) and 95% CIs. If various measurement techniques were employed, standardized MDs and 95% CIs were used. Statistical analyses were conducted using Review Manager (Version 5.4), with Forest plots and *I*^2^ statistics employed to detect inter-study heterogeneity. For *I*^2^ > 50%, indicative of substantial heterogeneity among the included studies, we employed the random-effects model to account for the variability. For *I*^2^ ≤ 50%, we used the fixed effects model. Substantial heterogeneity was further examined, and subgroup analyses were conducted for randomized controlled trials and specific patient subgroups.

## Results

### Literature search and studies included

A total of 9628 records were identified through our database search. Of these, 8750 were excluded because they are out of the scope of this analysis. This left 923 records for full-text assessment, from which 892 were excluded for various reasons, including incorrect setting, inappropriate intervention, and unavailability of full text. Consequently, 30 studies met the inclusion criteria. Of these, 15 focused solely on “admission avoidance HaH.” The remaining 15 studies that highlighted “early discharge HaH” were included in this review. Figure 1 demonstrates the selection flow.

Fifteen trials with a cumulative sample size of 4,190 patients were incorporated into this study. The trials involved patients with various health conditions, including cancer [13], acute medical conditions [5, 14–17], chronic medical conditions [4, 18, 19], orthopedic issues [18, 20–23], and medically complex conditions [18, 24, 25]. Of the 15 studies, 10 were trials related to early discharge HaH [4, 14, 15, 19–25]. Meanwhile, five trials studied HaH interventions that incorporated both early discharge and admission avoidance [15, 17, 18, 21, 24]. Seven studies employed a randomized controlled trial design, one used a prospective quasi-experiment [5], one was a prospective, non-randomized real-world cohort comparison [13], one was a retrospective study [22], and one was a quasi-experimental longitudinal study [21]. The characteristics of the included studies are shown in Additional file 1: Table S2. Meta-analysis related to early discharge HaH program in the past 20 years is shown in Additional file 1: Table S3.

### Quality of included studies

**Fig. 2:**
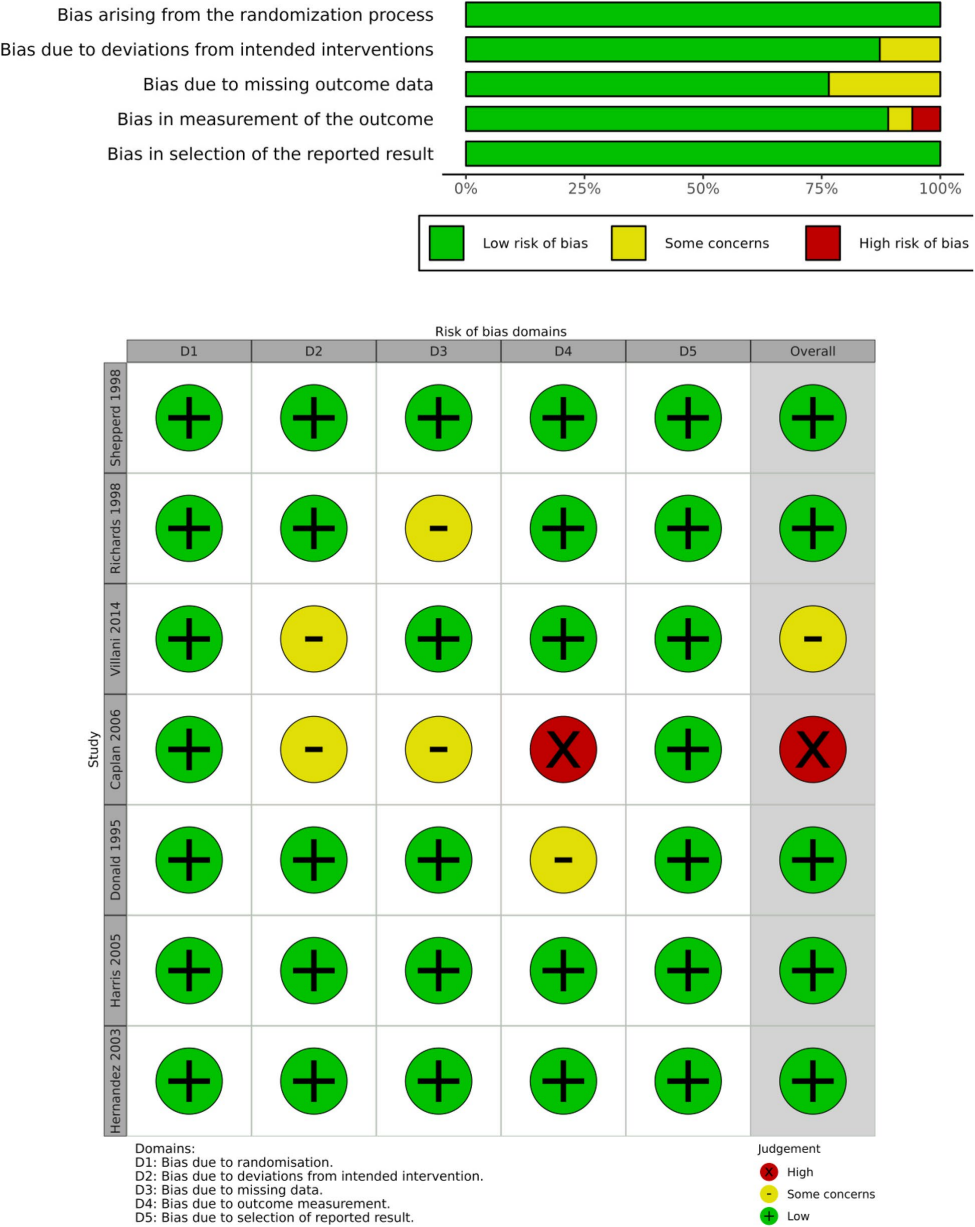
Risk of bias plots for RCT studies

**Fig. 3:**
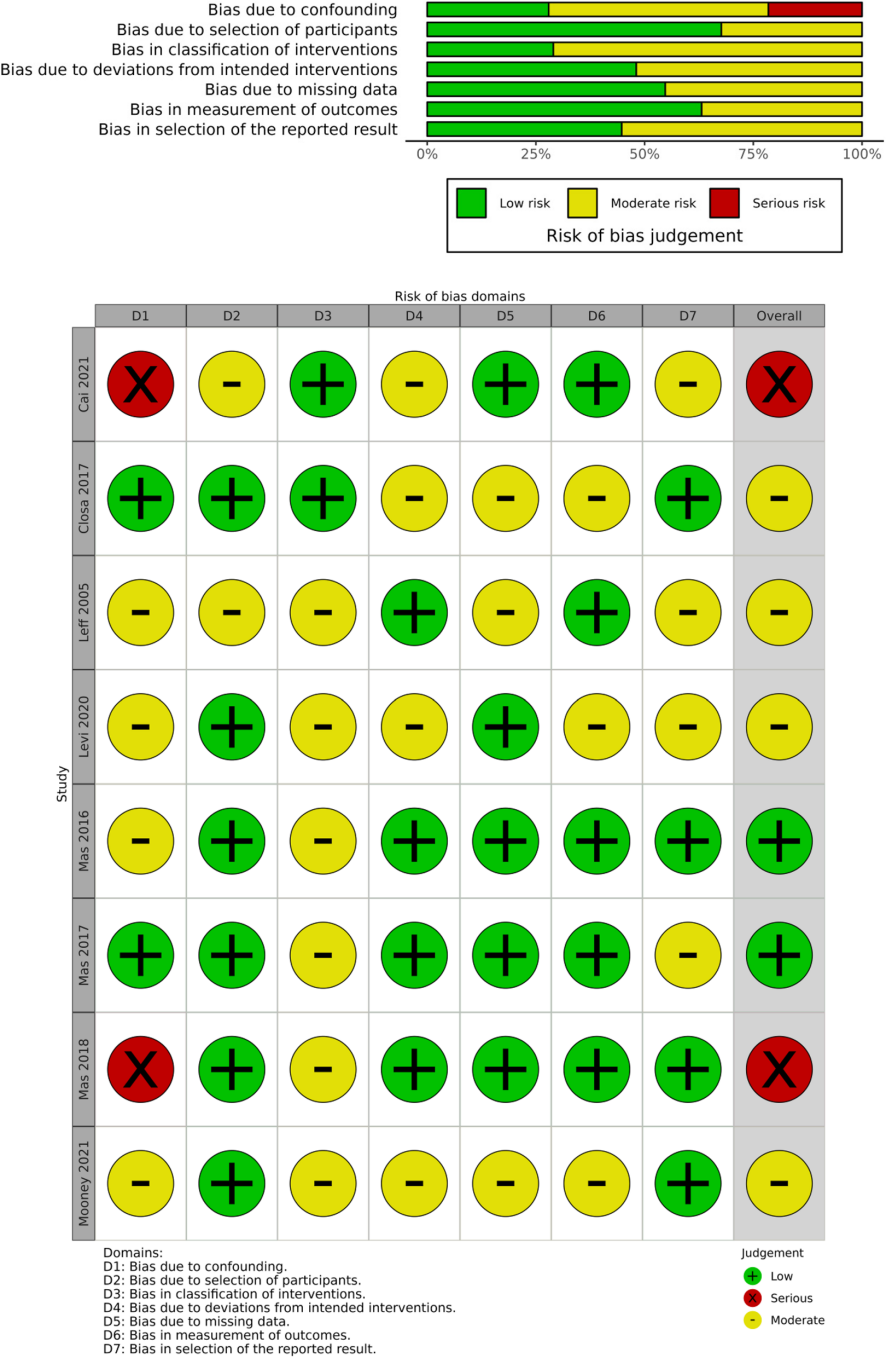
Risk of bias plots for non-RCT studies

The risk of bias for RCT and non-RCT studies is shown in Figs. 2 and 3. Results demonstrate that, although certain domains within some studies raised “some concerns,” the collective evaluation of these studies generally indicates a low risk of bias.

### Main analysis

#### Mortality

**Fig. 4:**
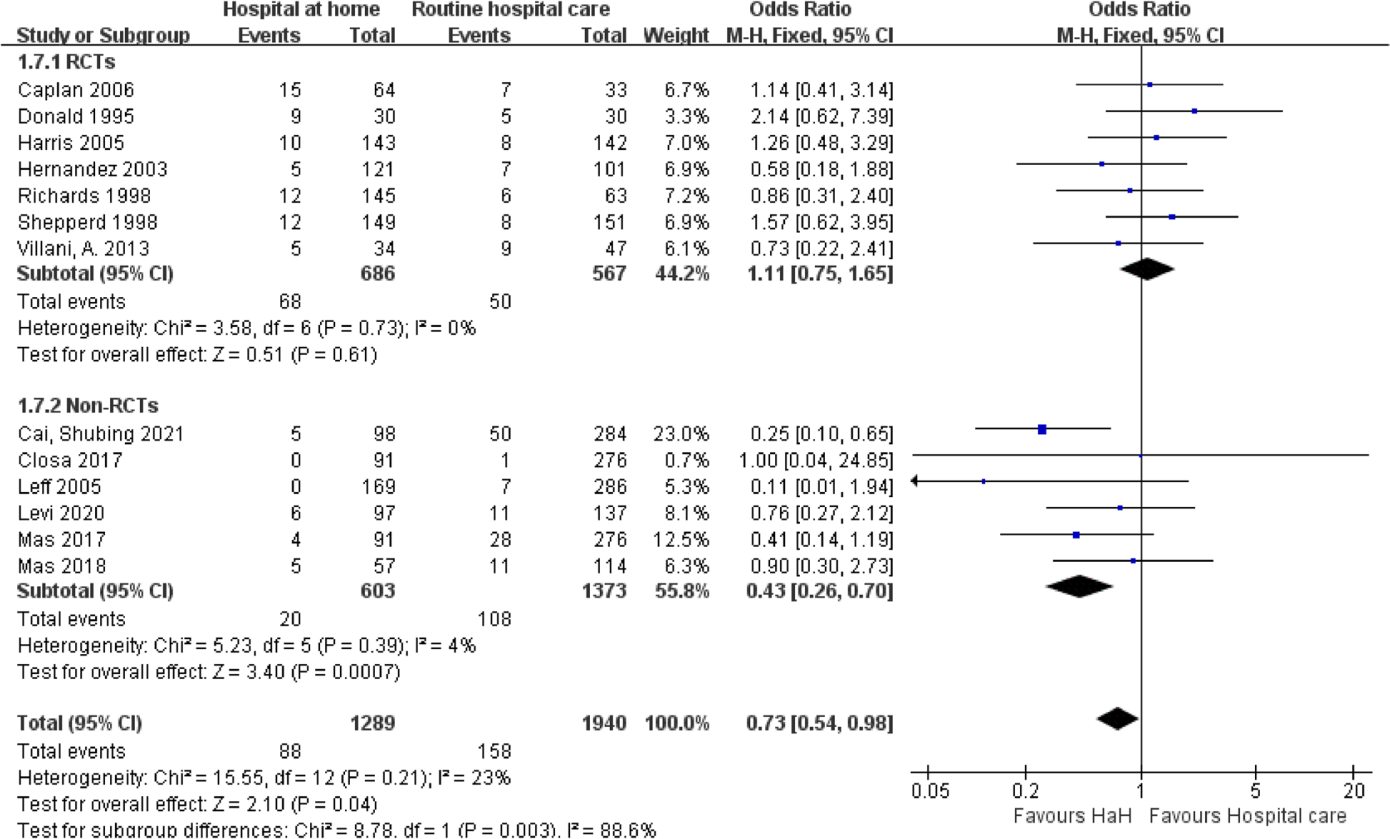
Early discharge hospital at home versus routine hospital care: mortality

The forest plot analysis for mortality outcomes comparing early discharge HaH programs to routine hospital care reveals a nuanced picture: RCTs show no significant difference between HaH and hospital care, with an odds ratio of 1.11 (95% CI: 0.75, 1.65) and minimal heterogeneity (*I*^2^ = 0%). Non-RCTs, however, indicate a significant mortality reduction in the HaH group (OR = 0.43; 95% CI: 0.26, 0.70), albeit with slightly more heterogeneity (*I*^2^ = 4%) (Fig. 4).

#### Readmission

**Fig. 5:**
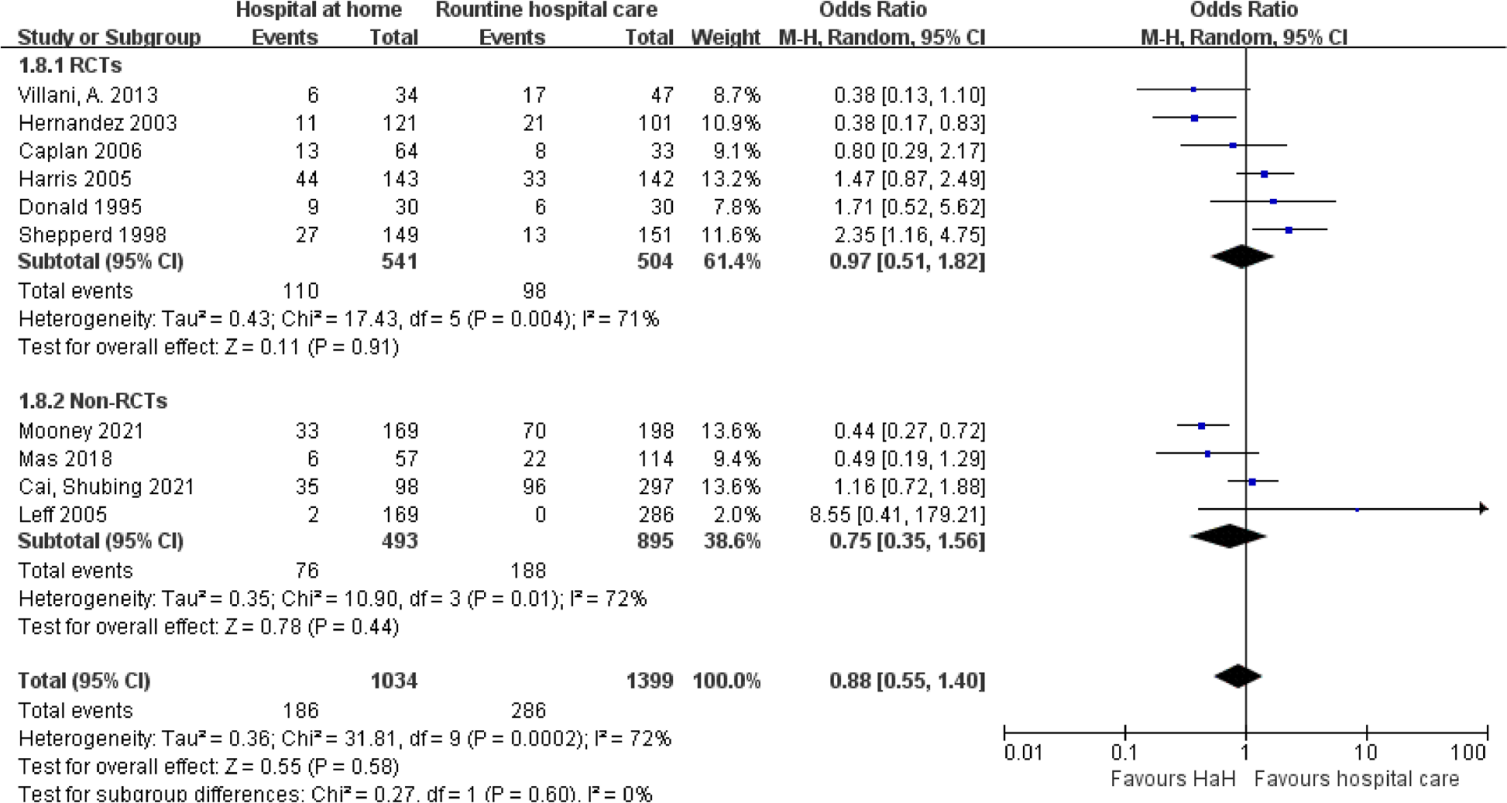
Early discharge hospital at home versus routine hospital care: readmission

The forest plot comparing early discharge HaH to routine hospital care shows no significant difference in readmission rates across both RCTs with an odds ratio of 0.97 (95% CI: 0.51, 1.82) and non-RCTs with an OR of 0.75 (95% CI: 0.35, 1.56), and this pattern holds in the combined analysis (OR 0.88; 95% CI: 0.55, 1.40). However, the high heterogeneity observed within both RCTs (*I*^2^ = 71%) and non-RCTs (*I*^2^ = 72%) suggests variability in the study outcomes, underscoring the need for cautious interpretation (Fig. 5).

#### Length of treatment

**Fig. 6:**
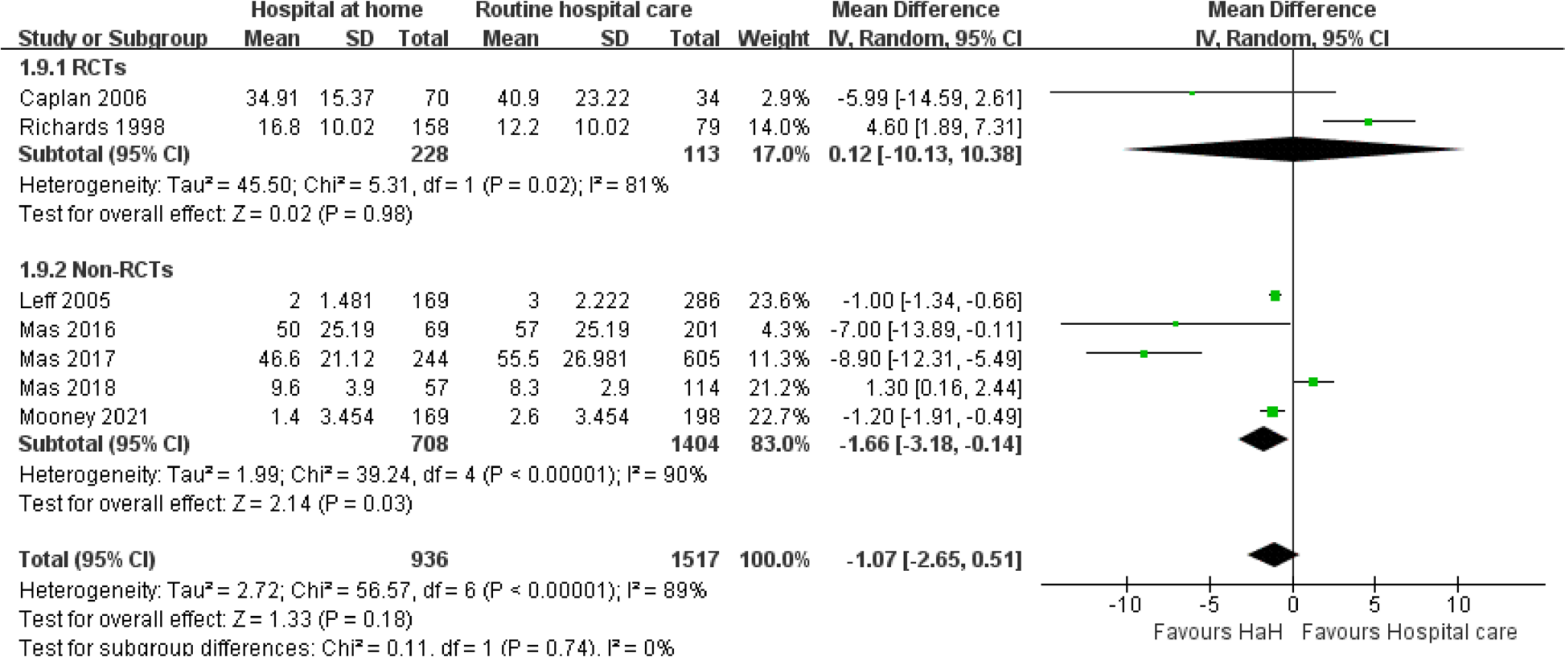
Early discharge hospital at home versus routine hospital care: length of treatment

The forest plot for length of treatment shows that in RCTs, the HaH group exhibits no significant difference compared to routine care, with a mean difference of 0.02 (95% CI: − 0.98 to 1.03) and low heterogeneity (*I*^2^ = 8%). In contrast, non-RCTs demonstrate a significant reduction in treatment duration for HaH with a mean difference of − 1.66 (95% CI: − 3.18 to − 0.14) but with substantial heterogeneity (*I*^2^ = 90%) (Fig. 6).

#### Functional status — Barthel index at the end up

**Fig. 7:**
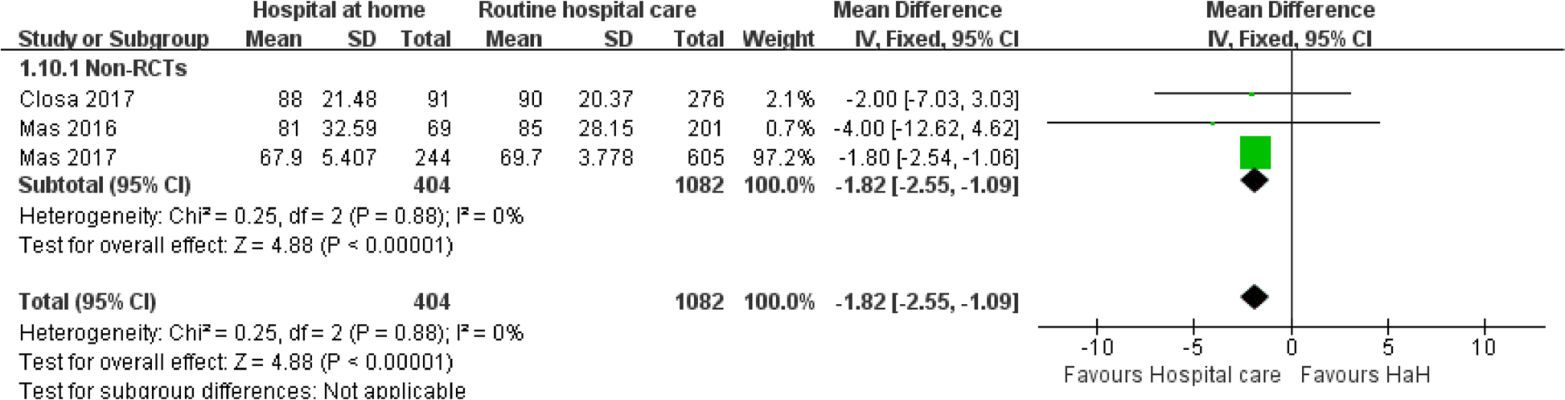
Early discharge hospital at home versus routine hospital care: functional status — Barthel index at the end up

Figure 7 indicates a notable reduction in treatment length with HaH when compared to standard hospital care, as shown by a mean difference of − 1.82 (95% CI: − 2.55, − 1.09) with negligible heterogeneity (*I*^2^ = 0%) in non-RCTs. A meta-analysis for RCTs on this outcome was not performed due to the presence of only a single study.

#### Mental status — MMSE

For the assessment of mental status using MMSE, our analysis considered one randomized controlled trial (RCT) [14] and one retrospective study [22]. Due to the presence of only one study in each category, we are not able to pool the results. The findings from both the retrospective study and the RCT indicated no significant difference in MMSE scores between the case group and the control group. Specifically, the retrospective study reported a mean difference of − 1.24 (95% CI: − 3.30 to 0.82, *N* = 235; *P* = 0.24), and the RCT showed a mean difference of − 2.59 (95% CI: − 5.45 to 0.27, *N* = 70; *P* = 0.08).

### Subgroup analyses based on disease classification

Subgroup analyses were conducted in patients with different medical conditions. Patients were classified into five groups: acute medical conditions [5, 14–17], chronic medical conditions [4, 18, 19], orthopedic conditions [18, 20–23], cancer [13], and medically complex conditions [18, 24, 25].

#### Mortality

**Fig. 8:**
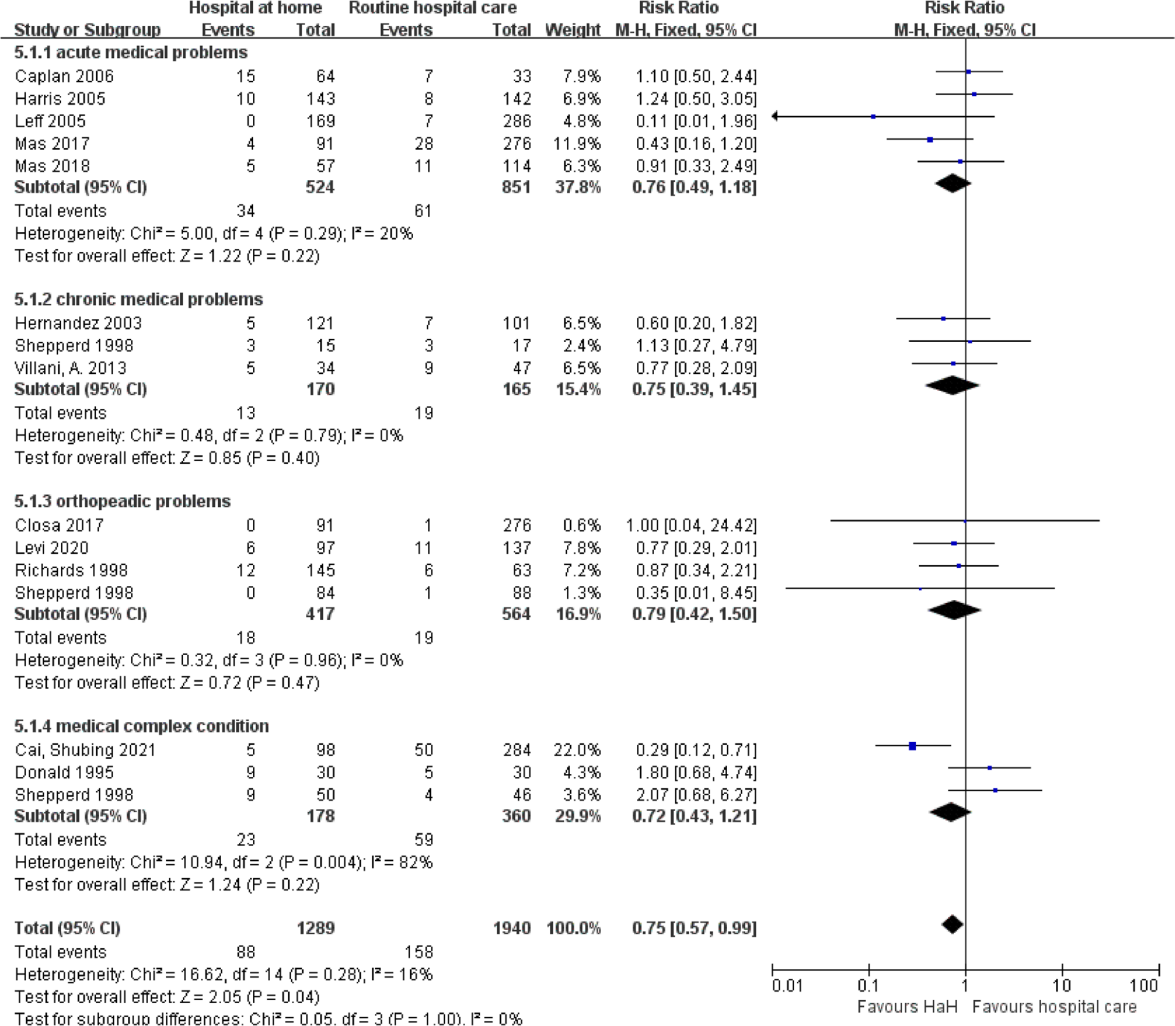
Early discharge hospital at home versus routine hospital care: subgroup analysis based on disease classification — mortality

Figure 8 reported the impact of early discharge HaH programs on mortality across various medical conditions. Cumulatively, the data indicates a significant 25% reduction in mortality risk for HaH participants (RR = 0.75, *P* = 1.00). Upon dissection by condition, both acute and chronic medical cases, as well as orthopedic and complex medical conditions, showed favorable trends towards HaH programs, even if individual disease categorizations did not always attain statistical significance. Notably, the relatively low heterogeneity among acute and chronic conditions (*I*^2^ = 20% and *I*^2^ = 0%, respectively) suggests consistent outcomes across these trials. The forest plot further reinforces these findings, with the majority of individual study outcomes leaning towards the early discharge HaH advantage and the aggregate result represented by the diamond, solidly positioned on the “early discharge HaH” side, underscoring the potential benefits of HaH in reducing mortality.

#### Readmission

**Fig. 9:**
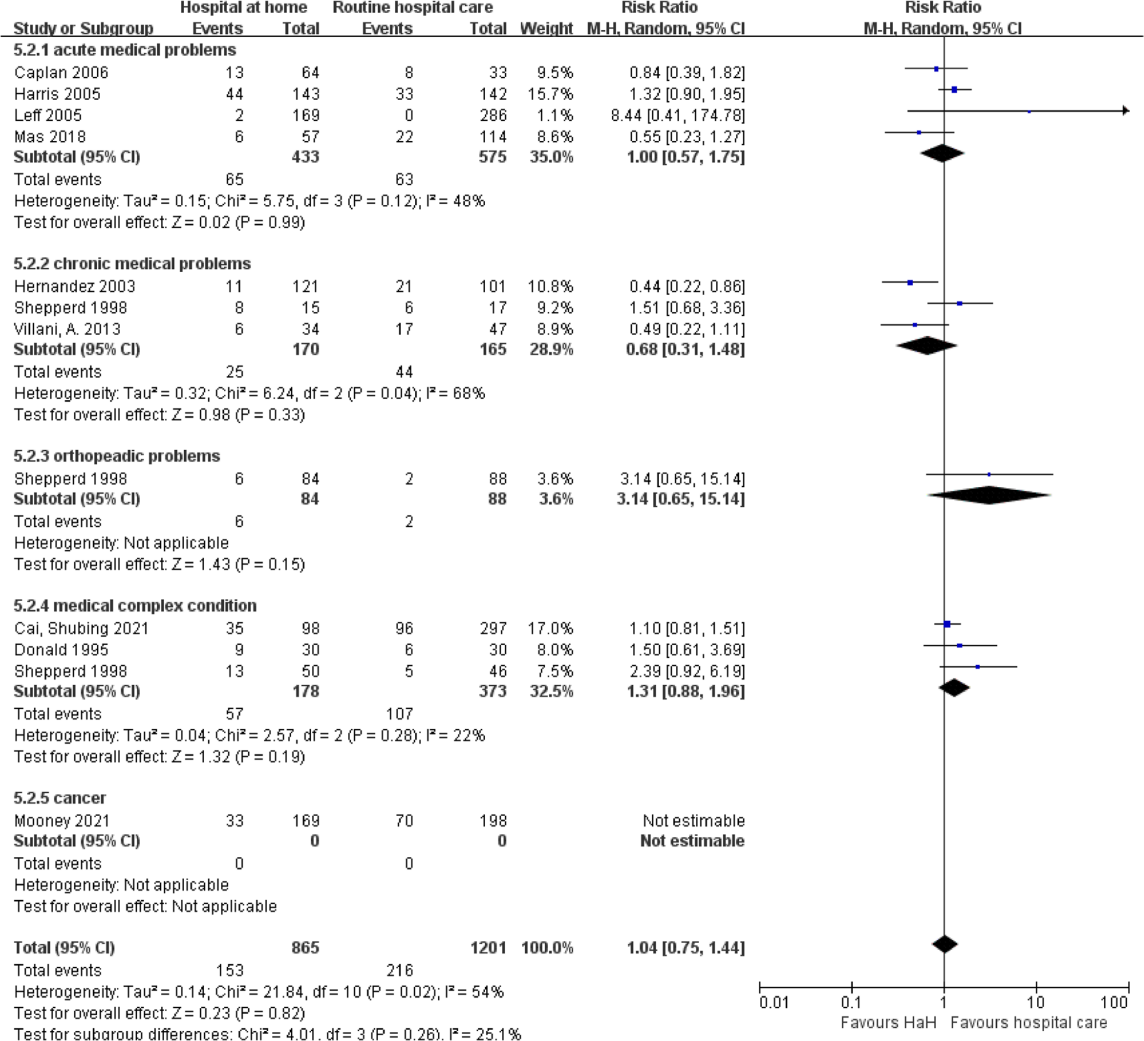
Early discharge hospital at home versus routine hospital care: subgroup analysis based on disease classification — readmission

Figure 9 illuminated the impact of early discharge HaH programs on readmission rates across diverse medical conditions. Overall, the combined data reveals a marginal increase in readmission risk for HaH programs, but the effect is statistically insignificant (RR = 1.04, *P* = 0.26). When segregating by disease type, acute medical cases depict an almost neutral effect, with a pooled risk ratio near unity (RR = 1.00). Chronic medical problems, while reflecting a slight reduction in readmission risk, also lacked statistical significance. Intriguingly, orthopedic cases displayed a notably higher readmission risk (RR = 3.14), though this observation was based on limited data and requires further substantiation. Medical complex conditions and cancer demonstrated neutral to increased risk, yet again without achieving statistical significance. An overarching observation is the pronounced heterogeneity in some disease categories, such as chronic medical problems (*I*^2^ = 68%), underscoring the importance of interpreting these results with caution. The forest plot bolsters these conclusions, with a mix of study outcomes on both sides of the neutrality line and the collective result, embodied by the diamond, tending slightly towards the “routine hospital” side. This suggests that while HaH may offer several advantages, it is crucial to consider patient-specific factors and disease categories when assessing its implications for readmission.

#### Length of treatment

**Fig. 10:**
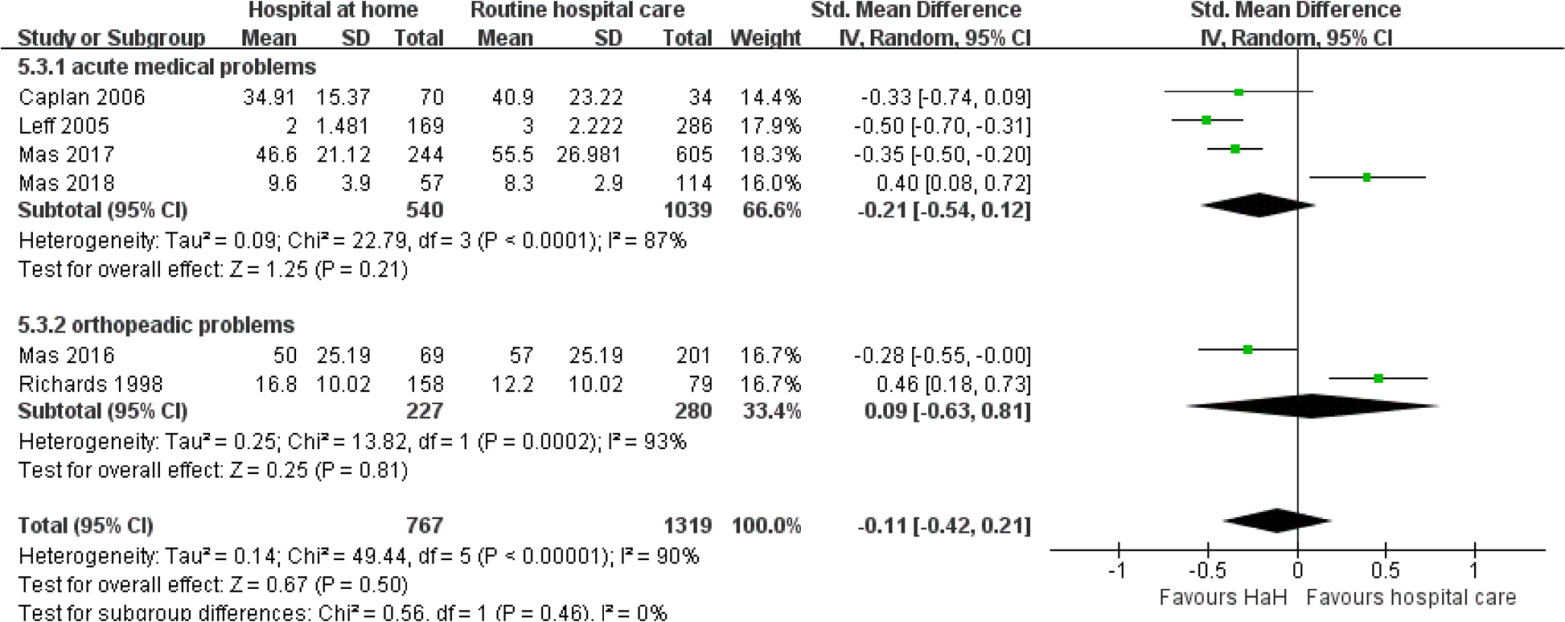
Early discharge hospital at home versus routine hospital care: subgroup analysis based on disease classification — length of treatment

Figure 10 elucidates the impact of early discharge HaH programs on the duration of treatment, quantified as the standard mean difference across various medical conditions. For acute medical problems, the pooled data indicates a modest reduction in treatment duration for HaH, albeit with a wide confidence interval that borders on the null effect (MDs =  − 0.21, *P* = 0.21). Notably, there is high heterogeneity (*I*^2^ = 87%), suggesting substantial variability among the included studies for this category. For orthopedic issues, although a reduction in treatment duration is observed for HaH, the confidence interval overlaps with zero, denoting potential insignificance in the effect (MDs =  − 0.09). The heterogeneity in this subgroup is also high (*I*^2^ = 93%), emphasizing the variability of study outcomes. The overall combined effect suggests a negligible reduction in treatment duration with HaH (MDs =  − 0.11), and the encompassing forest plot substantiates this with outcomes straddling both sides of the neutrality line. The prevailing observation is that while HaH may potentially reduce the duration of treatment, the extent of this reduction varies considerably across studies and disease categories. Given the high heterogeneity in results, careful interpretation and further research are necessary to delineate the specific scenarios where HaH offers significant time-saving benefits.

#### Barthel index at the end up

**Fig. 11:**
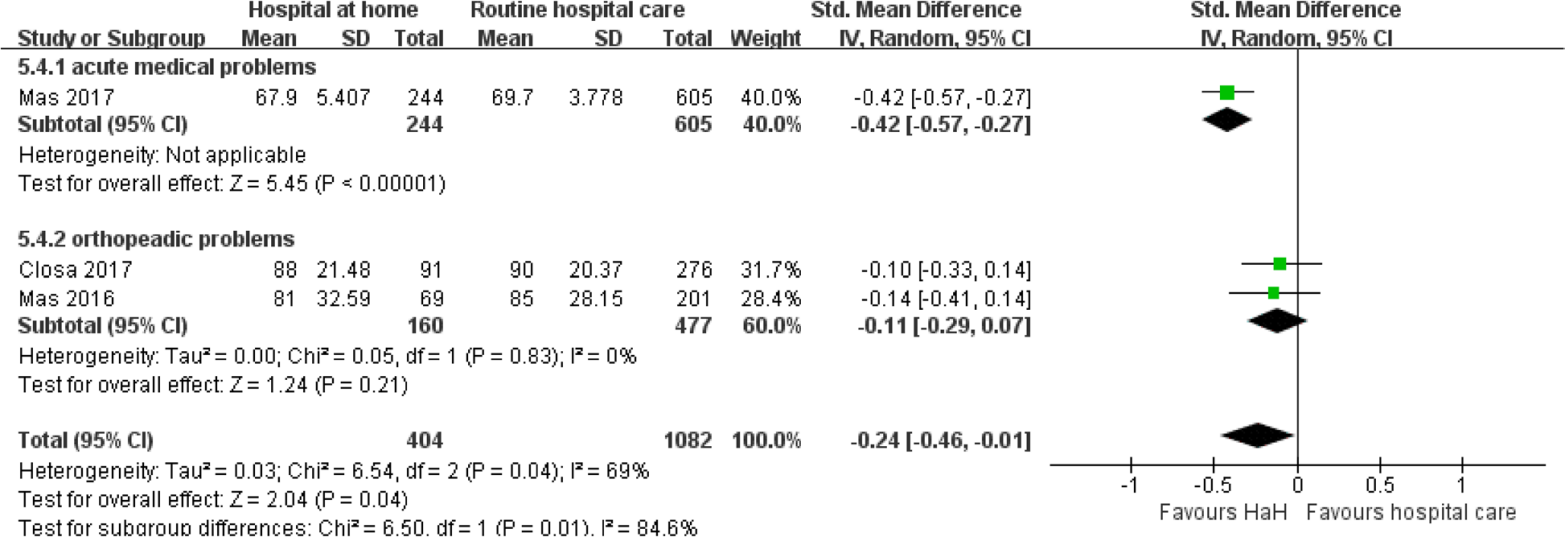
Early discharge hospital at home versus routine hospital care: subgroup analysis based on disease classification — Barthel index at the end up

Figure 11 conducted a subgroup analysis of the Barthel index scores at the endpoint, comparing early discharge HaH to traditional routine hospital care, segmented by acute medical problems and orthopedic conditions. For those with acute medical problems, HaH intervention suggests a significant reduction in Barthel index scores with a standard mean difference of − 0.42 (95% CI: − 0.57 to − 0.27), showcasing its potential efficacy. This result is statistically significant with a *p*-value of less than 0.00001. However, in orthopedic conditions, the results are less definitive. Although there is a trend towards a decrease in the Barthel index scores within the HaH group (MDs =  − 0.11), the confidence interval slightly spans the line of neutrality, indicating potential non-significance. This subgroup also displays moderate heterogeneity (*I*^2^ = 69%), hinting at diverse outcomes across the incorporated studies. In totality, while HaH seems to influence a reduction in Barthel index scores, the magnitude and robustness of this impact appear to be condition-specific. Collectively, these findings emphasize the potential benefits of HaH, particularly for patients with acute medical problems, but warrant cautious interpretation in the context of orthopedic issues.

## Discussion

Fifteen trials were incorporated into this systematic review examining early discharge HaH for older people. Our investigation encompassed a comprehensive exploration of the efficacy of early hospital discharge to home care across several key domains: mortality, readmission rates, duration of treatment, functional status (as quantified by the Barthel index), and cognitive health (as assessed by the MMSE).

Our analysis suggests that early discharge HaH correlates with a decrease in mortality rates, a conclusion drawn from 6 of the non-RCTs. In contrast, the impact of early discharge HaH on readmission rates appears negligible, a conclusion supported by four non-RCTs and five RCTs. The influence of early discharge HaH on cognitive health, based on MMSE scores, is similarly inconsequential, as demonstrated by one RCT and one non-RCT. Moreover, early discharge HaH might be linked to a decline in functional status, as gauged by the Barthel index, based on evidence from one RCT and three non-RCTs. After analyzing the data of the fifteen trials included, regardless of whether there was a statistically significant difference between the early discharge HaH group and the routine hospital care group from the forest map, the diamonds tended to be on the “early discharge HaH” side, emphasizing the role of early discharge HaH in mortality, readmission rate, length of treatment and Barthel index. Our subgroup analysis based on disease classifications—encompassing acute medical problems, chronic conditions, orthopedic issues, cancer, and medically complex conditions—revealed similar trends when compared with the main analysis.

In our analysis, the effectiveness for the same measures—such as mortality, length of treatment, and the Barthel index—between RCTs and non-RCTs is not always identical. This variance likely stems from the inherent differences in the design and execution of these two types of studies. RCTs, with their higher design rigor and controlled environments, minimize selection biases and provide stronger evidence of causality, while non-RCTs, reflecting broader and more diverse populations, may offer findings that are more generalizable but subject to inherent biases and variability. The observed discrepancy of effectiveness for the same measures between RCTs HaH and non-RCTs HaH highlights the importance of considering the real-world scenario in understanding reported intervention effectiveness.

The HaH model is distinguished by their more holistic approach compared to traditional hospital care, particularly for older adults. Its capacity to prevent the development of geriatric syndromes is achieved through the comprehensive method of care that addresses not only the medical but also the physical, emotional, and social needs of patients, thereby enhancing their overall well-being and reducing mortality risks.

Furthermore, the decline in functional status observed in some patients underscores the indispensability of incorporating a multidisciplinary team into the HaH model. Specifically, the integration of physiotherapists and occupational therapists is crucial. These professionals can tailor rehabilitation and daily activity programs to the individual needs of each patient, fostering not only the maintenance but potentially the improvement of functional status. Such an approach ensures that care is not merely reactive but preventive and rehabilitative, addressing both the immediate and long-term health needs of the older adults.

While our meta-analysis indicated that the efficacy of Hospital-at-Home (HaH) programs is equivocal with regard to readmission rates and certain cognitive and functional outcomes, the approach nevertheless offers several distinct advantages that warrant further exploration and development. One of the most salient advantages of HaH programs in the aftermath of the COVID-19 pandemic is the minimization of exposure to nosocomial infections for vulnerable older adults [26–28]. Traditional hospital settings, by their nature, expose patients to a variety of pathogens, posing an increased risk of acquiring secondary infections. This is especially critical for older adults who are often immunocompromised and thus more susceptible to infections. Implementing HaH programs can thus provide a safer, more controlled environment, reducing the likelihood of cross-infections. The healthcare industry’s experience in the pandemic underscores the urgency for such decentralized healthcare models that can offer quality care while mitigating risks associated with hospital-based treatment [29–31].

HaH programs could also play a pivotal role in bridging healthcare disparities observed in rural areas and developing countries, which frequently face infrastructural constraints and shortages of healthcare professionals. The burden of chronic diseases such as heart disease, cancer, diabetes, and mental disorders is high in low-income and middle-income countries and is expected to increase with population aging, urbanization, and globalization of risk factors [32, 33]. Implementing HaH in these settings may alleviate some of these challenges by providing essential healthcare services directly to the homes of older adults. This is particularly beneficial in geographical locations where distance and limited transportation options make it arduous for individuals to access healthcare facilities. HaH leverages telehealth technologies and portable medical equipment, making it a scalable solution that can extend the reach of quality healthcare to remote and underserved communities [34, 35]. While the effectiveness of HaH on specific clinical outcomes may remain under debate, these broader socio-medical benefits highlight the importance of its continued development and implementation. A comprehensive assessment of HaH programs should thus go beyond traditional clinical metrics to include factors like patient safety, accessibility, and equity, especially in the changing landscape of global healthcare [33].

Meanwhile, in this study, our analysis of functional status is limited to the Barthel index after the end of the trials, while ignoring the trend of changes in the Barthel index before and after the intervention treatment in both groups. Due to the different evaluation indicators used in different trials for the various states of patients before and after hospitalization, the emphasis on measurement indicators also varies. Some trials focus on measuring the index changes before and after admission, while others focus on measuring the Barthel index after discharge. These may reduce the accuracy of our assessment of the improvement in functional status between the early discharge group and the routine hospital care group. Moreover, the overlap of evaluation indicators for cognitive health in different experiments is also relatively low. If different indicators can be standardized, the evaluation in the mental state dimension will be more comprehensive.

## Conclusions

In summation, while early discharge HaH presents a viable approach for diminishing mortality and treatment duration, its efficacy remains ambiguous in relation to readmission, as well as cognitive and functional outcomes. Thus, a prudent interpretation of these findings is essential.

### Supplementary Information

Additional file 1: Table S1. PRISMA check list. Table S2. Characteristics of included studies. Table S3. Meta analysis on early discharge HaH program in the past 20 years. Text S1. Search Strategy Used In PUBMED.

## Data Availability

The data used to support the findings of this study are included within the article.

## Abbreviations

CI: Confidence intervals
COPD: Chronic obstructive pulmonary disease
ED HaH: Early discharge hospital at home
EQ-5D: The 5-level EQ-5D version
HaH: Hospital at Home
MD: Mean differences
MeSH: Medical Subject Headings
MMSE: The Mini-Mental State Examination
Non-RCTs: Non-randomized controlled trials
OR: Odds ratio
PRISMA: Preferred Reporting of Items for Systematic Reviews and Meta-Analyses
PROSPERO: International prospective register of systematic reviews
RCTs: Randomized controlled trials
RoB2: Version 2 of the Cochrane risk-of-bias tool for randomized trials
ROBINS-I: Risk Of Bias In Non-randomised Studies—of Interventions
Robvis: Risk of Bias VISualization
SF-36: The 36-Item Short Form Health Survey
