# Supplementary material for: Early discharge hospital at home as alternative to routine hospital care for older people: a systematic review and meta-analysis
Source: BMC Med. 2024 Jun 18;22:250. doi: 10.1186/s12916-024-03463-3 (PMC11184809; doi:10.1186/s12916-024-03463-3)
Supplement: Supplementary file 1 — Additional file 1: Table S1. PRISMA check list. Table S2. Characteristics of included studies. Table S3. Meta analysis on early discharge HaH program in the past 20 years. Text S1. Search Strategy Used In PUBMED. [file 12916_2024_3463_MOESM1_ESM.docx]

**Additional file 1**

Table S1. PRISMA check list

| **Topic** | **No.** | **Item** | **Location where item is reported** |
| --- | --- | --- | --- |
| **TITLE** |  |  |  |
| **Title** | 1 | Identify the report as a systematic review. | 1 |
| **ABSTRACT** |  |  |  |
| **Abstract** | 2 | See the PRISMA 2020 for Abstracts checklist | 2 |
| **INTRODUCTION** |  |  |  |
| **Rationale** | 3 | Describe the rationale for the review in the context of existing knowledge. | 3 |
| **Objectives** | 4 | Provide an explicit statement of the objective(s) or question(s) the review addresses. | 3 |
| **METHODS** |  |  |  |
| **Eligibility criteria** | 5 | Specify the inclusion and exclusion criteria for the review and how studies were grouped for the syntheses. | 4 |
| **Information sources** | 6 | Specify all databases, registers, websites, organisations, reference lists and other sources searched or consulted to identify studies. Specify the date when each source was last searched or consulted. | 4 |
| **Search strategy** | 7 | Present the full search strategies for all databases, registers and websites, including any filters and limits used. | 4 |
| **Selection process** | 8 | Specify the methods used to decide whether a study met the inclusion criteria of the review, including how many reviewers screened each record and each report retrieved, whether they worked independently, and if applicable, details of automation tools used in the process. | 5 |
| **Data collection process** | 9 | Specify the methods used to collect data from reports, including how many reviewers collected data from each report, whether they worked independently, any processes for obtaining or confirming data from study investigators, and if applicable, details of automation tools used in the process. | 6 |
| **Data items** | 10a | List and define all outcomes for which data were sought. Specify whether all results that were compatible with each outcome domain in each study were sought (e.g. for all measures, time points, analyses), and if not, the methods used to decide which results to collect. | 5 |
|  | 10b | List and define all other variables for which data were sought (e.g. participant and intervention characteristics, funding sources). Describe any assumptions made about any missing or unclear information. | 5 |
| **Study risk of bias assessment** | 11 | Specify the methods used to assess risk of bias in the included studies, including details of the tool(s) used, how many reviewers assessed each study and whether they worked independently, and if applicable, details of automation tools used in the process. | 7 |
| **Effect measures** | 12 | Specify for each outcome the effect measure(s) (e.g. risk ratio, mean difference) used in the synthesis or presentation of results. | 6 |
| **Synthesis methods** | 13a | Describe the processes used to decide which studies were eligible for each synthesis (e.g. tabulating the study intervention characteristics and comparing against the planned groups for each synthesis (item 5)). | 6 |
|  | 13b | Describe any methods required to prepare the data for presentation or synthesis, such as handling of missing summary statistics, or data conversions. | 6 |
|  | 13c | Describe any methods used to tabulate or visually display results of individual studies and syntheses. | 6 |
|  | 13d | Describe any methods used to synthesize results and provide a rationale for the choice(s). If meta-analysis was performed, describe the model(s), method(s) to identify the presence and extent of statistical heterogeneity, and software package(s) used. | 6 |
|  | 13e | Describe any methods used to explore possible causes of heterogeneity among study results (e.g. subgroup analysis, meta-regression). | 6 |
|  | 13f | Describe any sensitivity analyses conducted to assess robustness of the synthesized results. | 6 |
| **Reporting bias assessment** | 14 | Describe any methods used to assess risk of bias due to missing results in a synthesis (arising from reporting biases). | 7 |
| **Certainty assessment** | 15 | Describe any methods used to assess certainty (or confidence) in the body of evidence for an outcome. | 6 |
| **RESULTS** |  |  |  |
| **Study selection** | 16a | Describe the results of the search and selection process, from the number of records identified in the search to the number of studies included in the review, ideally using a flow diagram. | 7 |
|  | 16b | Cite studies that might appear to meet the inclusion criteria, but which were excluded, and explain why they were excluded. | 7 |
| **Study characteristics** | 17 | Cite each included study and present its characteristics. | 7 |
| **Risk of bias in studies** | 18 | Present assessments of risk of bias for each included study. | 8 |
| **Results of individual studies** | 19 | For all outcomes, present, for each study: (a) summary statistics for each group (where appropriate) and (b) an effect estimate and its precision (e.g. confidence/credible interval), ideally using structured tables or plots. | 9-15 |
| **Results of syntheses** | 20a | For each synthesis, briefly summarise the characteristics and risk of bias among contributing studies. | 9-15 |
|  | 20b | Present results of all statistical syntheses conducted. If meta-analysis was done, present for each the summary estimate and its precision (e.g. confidence/credible interval) and measures of statistical heterogeneity. If comparing groups, describe the direction of the effect. | 9-15 |
|  | 20c | Present results of all investigations of possible causes of heterogeneity among study results. | 9-15 |
|  | 20d | Present results of all sensitivity analyses conducted to assess the robustness of the synthesized results. | 9-15 |
| **Reporting biases** | 21 | Present assessments of risk of bias due to missing results (arising from reporting biases) for each synthesis assessed. | 15 |
| **Certainty of evidence** | 22 | Present assessments of certainty (or confidence) in the body of evidence for each outcome assessed. | 15 |
| **DISCUSSION** |  |  |  |
| **Discussion** | 23a | Provide a general interpretation of the results in the context of other evidence. | 15-17 |
|  | 23b | Discuss any limitations of the evidence included in the review. | 15-17 |
|  | 23c | Discuss any limitations of the review processes used. | 15-17 |
|  | 23d | Discuss implications of the results for practice, policy, and future research. | 15-17 |
| **OTHER INFORMATION** |  |  |  |
| **Registration and protocol** | 24a | Provide registration information for the review, including register name and registration number, or state that the review was not registered. | 4 |
|  | 24b | Indicate where the review protocol can be accessed, or state that a protocol was not prepared. | 4 |
|  | 24c | Describe and explain any amendments to information provided at registration or in the protocol. | 4 |
| **Support** | 25 | Describe sources of financial or non-financial support for the review, and the role of the funders or sponsors in the review. | 20 |
| **Competing interests** | 26 | Declare any competing interests of review authors. | 21 |
| **Availability of data, code and other materials** | 27 | Report which of the following are publicly available and where they can be found: template data collection forms; data extracted from included studies; data used for all analyses; analytic code; any other materials used in the review. | 21 |

Text S1. Search Strategy Used In PUBMED

We piloted our search in PubMed using the following search query and got 5,796 search results:

(("Home Care Services, Hospital-Based"[Mesh]) OR ("Home Care Services, Hospital-Based"[Title/Abstract]) OR ("home care"[Title/Abstract]) OR ("hospital based home care"[Title/Abstract]) OR ("home hospital"[Title/Abstract]) OR ("hospital at home"[Title/Abstract]) OR ("home health services"[Title/Abstract]) OR ("hospital in the home"[Title/Abstract])) AND (("elderly"[Title/Abstract]) OR ("older"[Title/Abstract]) OR ("aged"[Title/Abstract]) OR ("senior"[Title/Abstract]) OR ("geriatric"[Title/Abstract]) OR ("60 years"[Title/Abstract]) OR ("70 years"[Title/Abstract]) OR ("65 years"[Title/Abstract]) OR ("80 years"[Title/Abstract]) OR ("85 years"[Title/Abstract]))

Table S2. Characteristics of included studies

Richards 1998

| Study characteristics | |
| --- | --- |
| Methods | RCT |
| Participants | Location: UK  Population: patients recovering from hip replacement (N = 86), knee replacement (N = 86), and hysterectomy (N = 238); older adults medical patients (N = 96); and patients with chronic obstructive airways disease (N = 32)  Mean age (SD): T: 71 years (7.7); C: 70 years (8.7)  N = 300 (T = 149; C =151 ) |
| Interventions | Hospital at home (early discharge)  Health care service  Control group: routine hospital care  Study dates: October 1994 to November 1996 |
| Outcomes | Mortality; readmission; psychological status; quality of life; patient satisfaction; caregiver satisfaction; caregiver burden; cost  Follow up: 3 months |
| Notes |  |

Shepperd 1998

| Study characteristics | |
| --- | --- |
| Methods | RCT |
| Participants | Location: UK  Study population: mostly recovering form orthopaedic diseases  Median age (IQR): T: 79 years (72-84); C: 79 years (74-84)  N = 208 (T = 145; C = 63) |
| Interventions | Hospital at home (early discharge)  Health care service: 1 G grade district nurse coordinator, 1 E grade registered nurse, senior 1 physiotherapist, senior 1 occupational therapist, support workers, occupational therapy technician  Control group: routine hospital care  Study dates: July 1994 to October 1995 |
| Outcomes | Quality of life, satisfaction, and physical functioning assessed at 4 weeks and 3 months after randomization to treatment; length of stay in hospital and in hospital at home scheme after randomization; mortality at 3 months  Follow up: 4 weeks, 3 months |
| Notes |  |

Villani, A. 2013

| Study characteristics | |
| --- | --- |
| Study design | RCT |
| Participants | Location: Italy  Population: patients with chronic heart failure who were discharged from the hospital after an admission for a clinical rehabilitation  Mean age (SD): T: 71 years (4); C: 73 years (5)  N = 81 (T = 34; C = 47) |
| Interventions | Hospital at home (early discharge)  Health care service: the PDA acted as a reminder of the correct timing for the pills; at a fixed predefined time patients were asked to send via PDA their weight, blood  Control group: routine care from Heart Failure clinic  Study dates: not reported |
| Outcomes | Mortality; readmission; cost; psychological status; quality of life  Follow up: 12 months |
| Notes |  |

Cai, Shubing 2021

| Study characteristics | |
| --- | --- |
| Study design | Quality improvement study |
| Participants | Location: US  Population: veterans with medically complex conditions, primarily congestive heart failure and chronic obstructive pulmonary disease exacerbations  Mean age (SD): T: 67.7 years (1.14); C: 66.6 years (0.72)  N = 395 (T = 98; C = 297) |
| Interventions | Hospital at home (early discharge)  Health care service: a single daily RN visit, and provided a one-time physician visit, with daily physician oversight and addi_x005ftional physician visits if needed. Additional visits occurred at the discretion of the managing physician or at the request of the nurse  Control group: routine hospital care  Study dates: 2012 to 2018 |
| Outcomes | Hospital length of stay; 30-day and 90-day readmission; VA direct costs; combined VA and Medicare costs; mortality; 90-day nursing home use; days at home after hospital discharge  Follow up: 1 month and 3 months |
| Notes |  |

Caplan 2006

| Study characteristics | |
| --- | --- |
| Study design | RCT |
| Participants | Location: Australia  Population: all inpatients with a length of stay (LOS) exceeding 6 days, who were referred for geriatric rehabilitation  Mean age (SD): T: 83.86 years (7.80); C: 84.00 years (7.02)  N = 70 (T = 48; C = 22) |
| Interventions | Hospital at home (early discharge)  Health care service: early discharge rehabilitation at home  Control group: routine hospital care  Study dates: April 2000 to October 2002 |
| Outcomes | FIM; MMSE; geriatric depression score (GDS); satisfaction scores  Follow up: 1 month and 6 months |
| Notes |  |

Closa 2017

| Study characteristics | |
| --- | --- |
| Study design | Quasi-experimental longitudinal study |
| Participants | Location: Spain  Population: patients aged >65 years, attended by an acute orthopaedic surgery/traumatology unit after a fracture or arthroplasty  Mean age (SD): T: 82 years (7); C: 81 years (9)  N = 367 (T = 91; C = 276) |
| Interventions | Hospital at home (early discharge)  Health care service: managing the timely transfer from the acute hospital to the patient’s home and providing multi-disciplinary care from the first geriatrics rehabilitation team visit to the patient’s home  Control group: hospital-based postacute orthogeriatric care  Study dates: not reported |
| Outcomes | Degree of functional recovery; direct cost of care; resource savings resulting  from a reduction in acute hospital stay  Follow up: not reported |
| Notes |  |

Donald 1995

| Study characteristics | |
| --- | --- |
| Study design | RCT |
| Participants | Location: UK  Population: Patients admitted acutely under the care of the older adults Care Physicians were eligible for inclusion in the study if they lived within Gloucester City  Mean age (SD): T: 81.6 years (5.4); C: 84.0 years (6.0)  N = 60 (T = 30; C = 30) |
| Interventions | Hospital at home (early discharge)  Health care service: (i) intensive rehabilitation over a prolonged period as progress was made; (ii) a short period of rehabilitation with the provision of, and education with, appropriate aids; (iii) counselling and encouragement to regain confidence at home; (iv) little rehabilitation possible as motivation was low, or carer was content; (v) monitoring health  Control group: routine hospital care  Study dates: not reported |
| Outcomes | Barthel score; mental test score; categorization of mobility; continence on descriptive scales; Philadelphia Geriatric Center Morale score; description of dependency in six ADL tasks  Follow up: 4 weeks, 12 weeks, 26 weeks |
| Notes |  |

Harris 2005

| Study characteristics | |
| --- | --- |
| Study design | RCT |
| Participants | Location: New Zealand  Population: patients over being treated for an acute medical problem  Mean age: 80 years  N = 285 (T = 143; C = 142) |
| Interventions | Hospital at home (early discharge)  Health care service: on-call by a geriatrician; patient-centred planning; daily nursing review and adjustment of individual care plan; intensive home support with up to 24-h live-in home carer; professional multidisciplinary team support (occupational therapy, physiotherapy, social work); rehabilitation in the patient’s home; a discharge hand-over to ongoing support services  Control group: routine hospital care  Study dates: not reported |
| Outcomes | physical and mental function; self-rated recovery; SF-36; adverse events; readmission  Follow up: 10 days, 30 days, 90 days |
| Notes |  |

Hernandez 2003

| Study characteristics | |
| --- | --- |
| Study design | RCT |
| Participants | Location: Spain  Population: COPD exacerbation as a major cause of referral to the ER and absence of any criteria for imperative hospitalisation as stated by the British Thoracic Society (BTS) guidelines  Mean age (SD): T: 71.0 years (9.9); C: 70.5 years (9.4)  N = 222 (T = 121; C = 101) |
| Interventions | Hospital at home (early discharge)  Health care service: 1) an immediate or early discharge from the hospital was encouraged by the specialised team aiming to either avoid or reduce the length of inpatient hospitalisation; 2) a comprehensive therapeutic approach was tailored on an individual basis, according to the needs detected by the specialised team; and 3) patient support by a skilled respiratory nurse either through home visits or free-phone consultation was ensured during the 8-week follow-up period  Control group: routine hospital care  Study dates: November 1999 to November 2000 |
| Outcomes | risk factors for exacerbation; HRQL status; inpatient hospitalisations and/or ER admissions; clinical features of the current exacerbation; fragility factors  Follow up: 2 months |
| Notes |  |

Leff 2005

| Study characteristics | |
| --- | --- |
| Study design | Prospective quasi-experiment |
| Participants | Location: America  Population: COPD exacerbation as a major cause of referral to the ER and absence of any criteria for imperative hospitalisation as stated by the British Thoracic Society (BTS) guidelines  Mean age (SD): T: 71.0 years (9.9); C: 70.5 years (9.4)  N = 222 (T = 121; C = 101) |
| Interventions | Hospital at home (early discharge)  Health care service: 1) an immediate or early discharge from the hospital was encouraged by the specialised team aiming to either avoid or reduce the length of inpatient hospitalisation; 2) a comprehensive therapeutic approach was tailored on an individual basis, according to the needs detected by the specialised team; and 3) patient support by a skilled respiratory nurse either through home visits or free-phone consultation was ensured during the 8-week follow-up period  Control group: routine hospital care  Study dates: November 1999 to November 2000 |
| Outcomes | risk factors for exacerbation; HRQL status; inpatient hospitalisations and/or ER admissions; clinical features of the current exacerbation; fragility factors  Follow up: 2 months |
| Notes |  |

Levi 2020

| Study characteristics | |
| --- | --- |
| Study design | Retrospective study |
| Participants | Location: Israel  Population: patients 65 years or above who underwent surgical repair of a femoral neck fracture  Mean age (SD): T: 82.5 years (7.1); C: 80.2 years (8.9)  N = 235 (T = 97; C = 138) |
| Interventions | Hospital at home (early discharge)  Health care service: mobilization facilitated by all staff members, physiotherapy, occupational therapy, psychosocial intervention, preparation for discharge from the ward begins at the early stages of hospitalization, relevant community services to adjust living conditions, nursing help, and ongoing rehabilitation at home  Control group: routine hospital care  Study dates: January 1, 2016 to December 31, 2019 |
| Outcomes | Morbidity; medical status; functional status; rehabilitation time  Follow up: 6 months |
| Notes |  |

Mas 2016

| Study characteristics | |
| --- | --- |
| Study design | Quasi-experimental longitudinal study |
| Participants | Location: Spain  Population: Older patients with orthopaedic acute events were selected in the acute ward or after being attended in the Emergency Department  Median age (IQR): T: 83 years (79-87); C: 84 years (79-88)  N = 270 (T = 69; C = 201) |
| Interventions | Hospital at home (early discharge)  Health care service: managing needs of high risk patients by its specialized health staff using technology of the geriatric wards of the hospital, during the rehabilitation process. At home, complex conditions could be managed by specialist nurses  Control group: routine hospital care  Study dates: not reported |
| Outcomes | functional status; prevalence of delirium at admission; main geriatric syndromes  Follow up: 24 months |
| Notes |  |

Mas 2017

| Study characteristics | |
| --- | --- |
| Study design | Quasi-experimental longitudinal study |
| Participants | Location: Spain  Population: all the patients presented an acute medical or surgical condition leading to acute disability, and needing CGA-based hospital-based care  Median age (IQR): T: 83.8 years (82.9-84.6); C: 83 years (82.4-83.6)  N = 367 (T = 91; C = 276) |
| Interventions | Hospital at home (early discharge)  Health care service: managing needs of high risk patients by its specialized health staff using technology of the geriatric wards of the hospital, during the rehabilitation process. At home, complex conditions could be managed by specialist nurses  Control group: routine hospital care  Study dates: January, 2010 to December, 2012 |
| Outcomes | Health crisis resolution; functional resolution; favourable crisis resolution; length of intervention in days; BI at discharge, rehabilitation efficiency; discharge destination  Follow up: 36 months |
| Notes |  |

Mas 2018

| Study characteristics | |
| --- | --- |
| Study design | Quasi-experimental longitudinal study |
| Participants | Location: Spain  Population: older patients with chronic conditions attended at the emergency department or day hospital for an acute medical crisis  Mean age (SD): T: 84.3 years (7.6); C: 86.9 years (6.3)  N = 171 (T = 57; C = 114) |
| Interventions | Hospital at home (early discharge)  Health care service: managing needs of high risk patients by its specialized health staff using technology of the geriatric wards of the hospital, during the rehabilitation process. At home, complex conditions could be managed by specialist nurses  Control group: routine hospital care  Study dates: December, 2015 to July, 2016 |
| Outcomes | Health crisis resolution; functional resolution; favourable crisis resolution; length of intervention in days; BI at discharge, rehabilitation efficiency; discharge destination  Follow up: 1 month |
| Notes |  |

Mooney 2021

| Study characteristics | |
| --- | --- |
| Study design | Prospective, non-randomized, real-world cohort comparison |
| Participants | Location: America  Population: patients with cancer who were admitted by inpatient hospitalists’ referral to HH after hospitalization  Mean age (SD): T: 62.3 years (14.8); C: 61.7 years (14.1)  N = 367 (T = 169; C = 198) |
| Interventions | Hospital at home (early discharge)  Health care service: managing needs of high risk patients by its specialized health staff using technology of the geriatric wards of the hospital, during the rehabilitation process. At home, complex conditions could be managed by specialist nurses  Control group: routine hospital care  Study dates: December, 2015 to July, 2016 |
| Outcomes | Health crisis resolution; functional resolution; favourable crisis resolution; length of intervention in days; BI at discharge, rehabilitation efficiency; discharge destination  Follow up: 1 month |
| Notes |  |

Table S3. Meta analysis on early discharge HaH program in the past 20 years

| ID | Title | Year | Age stratification | Study design included | Participants |
| --- | --- | --- | --- | --- | --- |
| 1 | Early supported discharge services for stroke patients: a meta-analysis of individual patients' data | 2005 | No age stratification | RCTs | Older adults with a clinical diagnosis of stroke |
| 2 | Effectiveness of early discharge planning in acutely ill or injured hospitalized older adults: a systematic review and meta-analysis | 2013 | Age ≥ 65 years | RCTs and quasi-experimental trials | Older adults with acutely ill or injured hospitalized |
| 3 | Effectiveness of nurse-led early discharge planning programmes for hospital inpatients with chronic disease or rehabilitation needs: a systematic review and meta-analysis | 2015 | Age ≥65 years; Age ＜65 years | RCTs | Older adults with chronic disease or rehabilitation needs |
| 4 | Early Supported Discharge/Hospital At Home For Acute Exacerbation of Chronic Obstructive Pulmonary Disease: A Review and Meta-Analysis | 2016 | Age ≥ 75 years | RCTs | Older adults with acute exacerbation of COPD (AECOPD) |
| 5 | Early discharge compared with ordinary discharge after percutaneous coronary intervention - a systematic review and meta-analysis of safety and cost | 2017 | No age stratification | RCTs | Patients after percutaneous coronary intervention |
| 6 | Early supported discharge services for people with acute stroke | 2017 | No age stratification | RCTs | A selected older adults of stroke survivors with moderate disability |
| 7 | Early discharge hospital at home | 2017 | Age ≥ 18 years | RCTs | Patients who are eligible to received health care from an early discharge hospital at home service. |
| 8 | Safety of early discharge after primary angioplasty in low-risk patients with ST-segment elevation myocardial infarction: A meta-analysis of randomised controlled trials | 2018 | Age ≤ 75 years | RCTs | Older adults after primary angioplasty in low-risk patients with ST-segment elevation myocardial infarction |
| 9 | Early Versus Standard Discharge After Transcatheter Aortic Valve Replacement: A Systematic Review and Meta-Analysis | 2018 | No age stratification | Observational studies | Patients after Transcatheter Aortic Valve Replacement |
| 10 | Next-Day Discharge vs Early Discharge After Transcatheter Aortic Valve Replacement: Systematic Review and Meta-Analysis | 2022 | No age stratification | Observational studies | Patients after Transcatheter Aortic Valve Replacement |
| 11 | Early supported discharge for older adults admitted to hospital with medical complaints: a systematic review and meta-analysis | 2022 | Age＞65 years | RCTs and quasi-experimental trials | Older adults admitted to hospital with medical complaints |
| 12 | Early supported discharge for older adults admitted to hospital with medical complaints: a systematic review and meta-analysis | 2023 | No age stratification | RCTs and quasi-RCTs | Older adults admitted to hospital with medical complaints |
| 13 | Early supported discharge for older adults admitted to hospital after orthopaedic surgery: a systematic review and meta-analysis | 2024 | Age ≥ 65 years | RCTs and quasi-RCTs | Older adults who were admitted to the acute care setting for orthopaedic complaints |

Utilizing 'early discharge' as our primary search term, we identified 13 review articles on early discharge Hospital at Home programs through a PubMed search. Our meta-analysis distinguishes itself from earlier studies in four aspects: it incorporates recent evidence, zeroes in on the older adult population, includes a mix of RCTs and observational studies, and provides a comprehensive examination before delving into disease-specific analyses, diverging from prior reviews that might focus solely on specific HaH program diseases.
